# *SLIT2* promoter methylation analysis in neuroblastoma, Wilms' tumour and renal cell carcinoma

**DOI:** 10.1038/sj.bjc.6601447

**Published:** 2004-01-20

**Authors:** D Astuti, N F da Silva, A Dallol, D Gentle, T Martinsson, P Kogner, R Grundy, T Kishida, M Yao, F Latif, E R Maher

**Affiliations:** 1 Section of Medical and Molecular Genetics, Department of Paediatrics and Child Health, University of Birmingham, The Medical School, Edgbaston, Birmingham B15 2TT, UK; 2 Cancer Research UK Renal Molecular Oncology Research Group, University of Birmingham, The Medical School, Edgbaston, Birmingham B15 2TT, UK; 3 Department of Clinical Genetics, Gothenburg University, Sahlgrenska University Hospital/Ostra, S-416 85 Gothenburg, Sweden; 4 Childhood Cancer Research Unit, Department of Women and Child Health, Karolinska Institute, Karolinska Hospital, S-171 76 Stockholm, Sweden; 5 Department of Paediatric Oncology, Birmingham Children's Hospital, UK; 6 Yokohama City University School of Medicine, Yokohama, Japan

**Keywords:** neuroblastoma, Wilms' tumour, renal cell carcinoma, epigenetics, *SLIT2*

## Abstract

The 3p21.3 *RASSF1A* tumour suppressor gene (TSG) provides a paradigm for TSGs inactivated by promoter methylation rather than somatic mutations. Recently, we identified frequent promoter methylation without somatic mutations of *SLIT2* in lung and breast cancers, suggesting similarities between *SLIT2* and *RASSF1A* TSGs. Epigenetic inactivation of *RASSF1A* was first described in lung and breast cancers and subsequently in a wide range of human cancers including neuroblastoma, Wilms' tumour and renal cell carcinoma (RCC). These findings prompted us to investigate *SLIT2* methylation in these three human cancers. We analysed 49 neuroblastomas (NBs), 37 Wilms' tumours and 48 RCC, and detected *SLIT2* promoter methylation in 29% of NB, 38% of Wilms' tumours and 25% of RCC. Previously, we had demonstrated frequent *RASSF1A* methylation in the same tumour series and frequent *CASP8* methylation in the NB and Wilms' tumour samples. However, there was no significant association between *SLIT2* promoter methylation and *RASSF1A* or *CASP8* methylation in NB and RCC. In Wilms' tumour, there was a trend for a negative association between *RASSF1A* and *SLIT2* methylation, although this did not reach statistical significance. No associations were detected between *SLIT2* promoter methylation and specific clinicopathological features in the tumours analysed. These findings implicate *SLIT2* promoter methylation in the pathogenesis of both paediatric and adult cancers and suggest that further investigations of *SLIT2* in other tumour types should be pursued. However, epigenetic inactivation of *SLIT2* is less frequent than *RASSF1A* in the tumour types analysed.

Methylation of CpG dinucleotides in the promoter regions of tumour suppressor genes (TSGs) producing transcriptional silencing is a major mechanism of TSG inactivation in many human cancers (Baylin *et al*, 1998; Jones, 1999; Tycko, 2000; Costello and Plass, 2001). The frequency of TSG inactivation by *de novo* methylation varies between TSGs and between cancers. Initially, epigenetic silencing of TSGs was reported as an alternative, but a minor mechanism of inactivation for TSGs such as *RB1* and *VHL* (Greger *et al*, 1994; Herman *et al*, 1994). However, recently a new class of TSGs has been recognised for which epigenetic silencing is the overwhelming mechanism of inactivation and somatic mutations are rare. This paradigm is exemplified by the *RASSF1A* 3p21.3 TSG, which is methylated in a wide range of human cancers (Dammann *et al*, 2000; Agathanggelou *et al*, 2001; Astuti *et al*, 2001; Burbee *et al*, 2001; Dreijerink *et al*, 2001; Morrissey *et al*, 2001).

Recently, we identified frequent promoter region hypermethylation of *SLIT2* in lung and breast cancers (Dallol *et al*, 2002a). *SLIT2* maps to 4p15.2 and encodes a human orthologue of the *Drosophila* Slit protein, a secreted glycoprotein, which regulates axon guidance, branching and neural migration during development of the central nervous system (Brose *et al*, 1999). Slit exerts its effects as a diffusible chemorepellent via its receptor Roundabout (Robo) (Stein and Tessier-Lavigne, 2001). In mammals, three distinct slit genes (*slit1*, *slit2*, and *slit3*) and three distinct robo genes (*robo1*, *robo2*, *rig-1*) have been identified. In lung and breast cancers, *SLIT2* mutations were not detected despite frequent promoter methylation (similar to *RASSF1A*) (Dallol *et al*, 2002a). Methylation of *RASSF1A* was first reported in lung and breast cancers, but subsequently we and others demonstrated *RASSF1A* methylation in renal cell carcinoma (RCC) and in common childhood tumours such as neuroblastoma (NB) and Wilms' tumour (Astuti *et al*, 2001; Dreijerink *et al*, 2001; Lo *et al*, 2001; Morrissey *et al*, 2001; Wagner *et al*, 2002). These findings prompted us to extend our studies of *SLIT2* inactivation by studying *SLIT2* promoter methylation in NB, Wilms' tumour and RCC.

## MATERIALS AND METHODS

### Patients and samples

A total of 134 tumour samples were analysed (49 NBs, 37 primary Wilms' tumours and 48 adult RCC). Details of the tumours have been published previously (Astuti *et al*, 2001; Wagner *et al*, 2002). DNA was extracted from tumour and normal tissues (blood or matched kidney) by standard methods.

### Sodium bisulphite modification

Sodium bisulphite modification was carried out using an adapted method (Herman *et al*, 1996). Genomic DNA (0.5–1 *μ*g) was denatured at 37°C for 10 min in 0.3 M NaOH. Unmethylated cytosines were sulphonated by incubation in 3.12 M sodium bisulphite/1 M hydroquinone (pH 5) at (95°C (30 s) 50°C (15 min)) × 20 cycles. The resulting sulphonated DNA was purified using the Wizard DNA clean-up system (Promega, Southampton, UK), according to the manufacturer's instructions, except that DNA was eluted with distilled water (50 *μ*l) at room temperature. Following elution, DNA was desulphonated in 0.3 M NaOH for 5 min at room temperature, then the DNA was precipitated with NaOAc (5 *μ*l of 3 M) and ethanol (125 *μ*l of 100%) overnight at −20°C and resuspended in 50 *μ*l distilled water.

### Methylation-specific polymerase chain reaction (PCR)

The CpG island within the putative *SLIT2* promoter has been described in detail previously (Dallol *et al*, 2002a). Methylation Specific PCR (MSP) analysis was performed essentially using primers and conditions as described in Dallol *et al*, 2002a). Combined Bisulphite Restriction Analysis (CoBRA) analysis was carried out as follows. In brief, the putative promoter region from nt −761 to −212 (relative to the translation start site) was amplified with primers reported previously (*Slit2*MODF-(5′-GGGAGGTGGGATTGTTTAGATATTT-3′ and *Slit2*MODR2 (5′-CAAAAACTCCTTAAACAACTTTAAATCCTAAAA-3′). From this reaction, one out of 50 of the volume was used as a template for a nested PCR with primers *Slit2*MODF as above and *Slit2*MODR (5′-ACTAAAACTTCCAACAACTACTAAAATACAAAAA-3′) to produce a 418 bp product (PCR conditions: 95°C for 10 min, followed by 30–40 cycles of 1 min denaturation at 95°C, 1 min annealing at 54°C, and 2 min extension at 74°C). PCR products were then digested by *Bst*U1 restriction enzyme to assess the methylation status of samples.

### Sequencing of PCR products

MSP and CoBRA products were excised from agarose gels and extracted using the QIAquick Gel Extraction Kit (Qiagen, Crawley, UK, West Sussex), according to the manufacturer's instructions. Products were confirmed by direct sequencing from the forward PCR primer using ABI Prism® BigDye™Terminators Cycle Sequencing Kit according to the manufacturer's instructions and run using ABI 377 automatic sequencers.

### 5-aza-2′-deoxycytidine treatment of cell lines

5-aza-2′-deoxycytidine (5-aza-dC, Sigma, Poole, UK) was freshly prepared in ddH_2_O (at 2 mg ml^−1^) and filter-sterilised. A total of 0.5–1 × 10^6^ cells were plated in 75 cm^2^ flask in RPMI 1640 media supplemented with 10% foetal calf serum and left to settle for 24 h (day 0). Kidney and NB cell lines were treated with 2 *μ*M 5-aza-dC for a total of 5 days. Kidney and NB have a different rate of growth. Kidney tumour cell lines were treated with 5-aza-dC on days 1, 3 and 5 and harvested on day 6, with a medium change in days 2 and 4 (Nojima *et al*, 2001). Neuroblastoma cell lines were treated on days 1 and 4. The medium was changed 24 h after treatment and then every 3 days. RNA was prepared after treatment using the RNeasy kit (Qiagen) according the manufacturer's guidelines. *SLIT2* gene expression was ascertained by RT–PCR using the primers 5′-GGTGTCCTCTGTGATGAAGAG-3′ and 5′-GTGTTTAGGAGACACACCTCG-3′, resulting in a product size 387 bp. As a control, the GAPDH primers used were: 5′-GACCCCTTCATGACCTCAACTACA-3′ and 5′-CTAAGCAGTTGGTGGTGCAGGA-3′, resulting in a PCR product of 369 bp.

### Microsatellite repeat analysis – loss of heterozygosity

By searching GDB and the UCSC assembly of the human genome sequence, we identified D4S1546 as the closest marker to *SLIT2* (within 100 kb). A 4p15.2 allele loss was assessed with the D4S1546 marker. (PCR conditions: 95°C for 5 min followed by 35 cycles of 95°C for 30 s, 52°C (55°C) for 30 s, and 72°C for 30 s and a final extension of 10 min at 72°C).

### Statistical analysis

Fisher's exact test was used as appropriate. *P*-values of <0.05 were considered to be statistically significant.

## RESULTS

### *SLIT2* methylation status in NB

**Figure 1:**
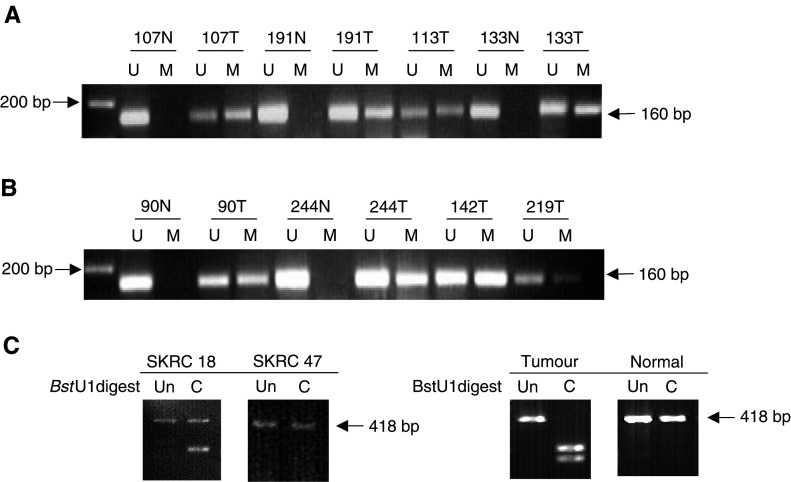
Methylation analysis of *SLIT2* by MSP in neuroblastoma tumours (**A**) and Wilm's tumours (**B**) and by CoBRA in RCC cell lines and primary kidney tumours and corresponding normal tissue (**C**). (**A** and **B**). Bisulphite-modified DNA was amplified with primers specific for unmethylated (U) and methylated (M) DNA. Tumours positive for *SLIT2* methylation are depicted by the presence of a 160 bp product when using specific primers for methylated DNA. N=blood; T=tumour tissue. (**C**) Bisulphite-modified DNA was amplified by nested PCR and then digested with *Bst*U1 restriction enzyme for 4 h at 60°C, uncut (Un) and cut (C). SKRC 18 is partially methylated while SKRC 47 is unmethylated. The RCC tumour shown above is completely methylated.

*SLIT2* promoter methylation status was analysed in 49 primary NB tumours and 29% (14 out of 49) demonstrated *SLIT2* CpG island promoter methylation (Figure 1A

). Promoter CpG island methylation was confirmed by direct sequencing of five clones from one tumour (Figure 4B). We also analysed eight NB cell lines for *SLIT2* methylation by restriction digestion and two cell lines (SK-N-F1 and SK-N-SH) were found to be partially methylated. *SLIT2* methylation was detected in one out of 49 corresponding normal blood samples.

**Figure 2:**
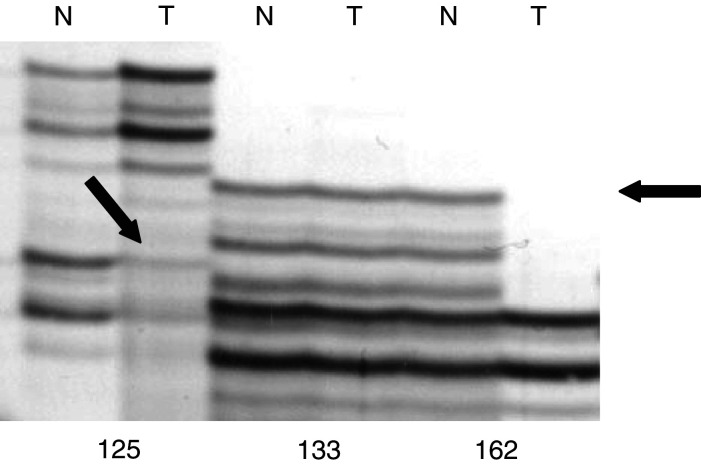
Genotyping of marker D4S1546 in neuroblastoma tumours. N and T, matched DNA samples from blood (N) and tumour tissue (T). Tumours 125 and 162 showed LOH, while tumour 133 shows retention of allele. The position of the lost allele is indicated by the arrows.

To investigate the 4p15.2 allelic status of NBs with *SLIT2* methylation, we typed 13 methylated tumours for loss of heterozygosity (LOH) at D4S1546 that maps close to *SLIT2*. In all, 33% (four out of 11) of informative tumours demonstrated allele loss consistent with homozygous *SLIT2* inactivation (Figure 2

).

#### *SLIT2* promoter methylation is associated with transcriptional silencing

**Figure 3:**
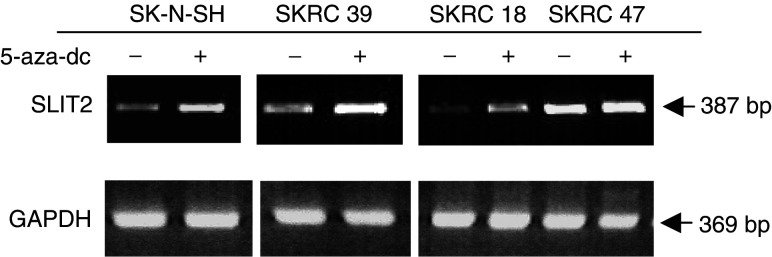
*SLIT2* expression by RT–PCR in neuroblastoma (SK-N-SH) and renal cell carcinoma cell lines (SKRC 39, SKRC 18 and SKRC 47), without (−) and with (+) 5-aza-2′-deoxycytidine (5-aza-dc) treatment. Cells were treated for up to 5 days with 5-aza-dc and expression was analysed by RT–PCR. The methylated cell lines (SK-N-SH, SKRC 39, SKRC 18) show an increase in *SLIT2* expression after 5-aza-dc treatment, while the unmethylated cell line SKRC 47 presents no changes in *SLIT2* expression before and after 5-aza-dc treatment.

To determine the relationship of *SLIT2* promoter region CpG island methylation and *SLIT2* transcript expression in the NB cell lines SK-N-F1 and SK-N-SH, we treated the cells with the demethylating agent, 5-aza-dC, for 5 days. The 5-aza-dC treatment significantly increased *SLIT2* expression in both cell lines, but there was little or no change in the expression of *GAPDH* expression after the 5-aza-dC treatment (Figure 3

).

#### *SLIT2*, *CASP8* and *RASSF1A* methylation status in NB

Previously, we reported that *RASSF1A* and caspase 8 (*CASP8*) promoter methylation occurred in 55 and 40% of NBs, respectively. To determine whether there was any relationship between *SLIT2* promoter methylation and *de novo* methylation of *RASSF1A* and *CASP8* promoters, we compared the frequencies of *RASSF1A* and *CASP8* methylation in tumours with and without *SLIT2* methylation (using previously published *RASSF1A* and *CASP8* methylation data (Astuti *et al*, 2001). *CASP8* methylation was detected in 36% of *SLIT2* methylated and 41% *SLIT2* unmethylated tumours (*P*=1.0). *RASSF1A* promoter methylation was more frequent in tumours with *SLIT2* promoter methylation (77 *vs* 59%), but this did not reach statistical significance (*P*=0.32).

#### Neuroblastoma clinicopathological features and *SLIT2* methylation status

We compared the results of *SLIT2* methylation status in our tumour series to the previously reported results for allelic loss of 1p or 3p loss, N-myc amplification and 17q gain (Martinsson *et al*, 1997). There was no correlation between *SLIT2* methylation and 1p allele loss (22% of *SLIT2* methylated and 22% of unmethylated tumours, *P*=1.0), 3p allele loss (25 *vs* 12%, *P*=0.57), 17q gain (50 *vs* 65%, *P*=0.62) or N-myc amplification status (15 *vs* 13%, *P*=1.0). There was no association between tumour stage and *SLIT2* methylation status: *SLIT2* methylation was present in 33% of stage 1, 2 and 4S tumours and in 26% of stage 3 and 4 tumours (*P*=0.73).

### Methylation analysis of *SLIT2* in primary Wilms' tumours

**Figure 4:**
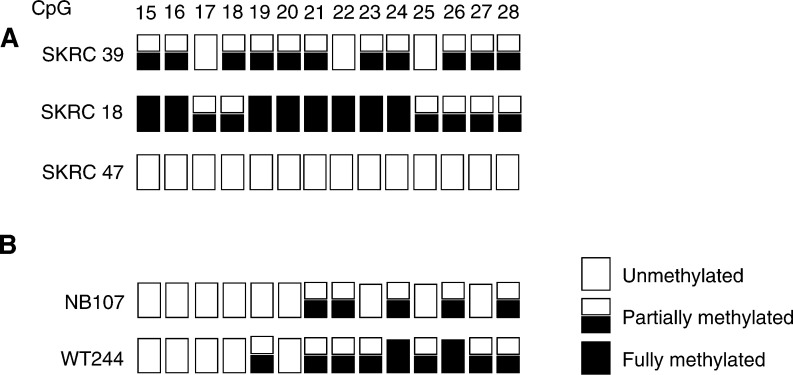
*SLIT2* methylation profile. Illustration of the *SLIT2* methylation pattern detected in (**A**) kidney tumour cell lines (SKRC 39, SKRC 18 and SKRC 47) and (**B**) neuroblastoma tumours (NB107) and Wilm's tumours (WT244). The CpG island numbered according to Dallol *et al* (2002a).

Next, we proceeded to analyse *SLIT2* promoter methylation status in 37 Wilms' tumours that had been investigated previously for *RASSF1A* and *CASP8* promoter methylation status (Wagner *et al*, 2002 and unpublished observations). In total, 38% (14 out of 37) Wilms' tumours demonstrated *SLIT2* CpG island promoter methylation (Figure 1B). Promoter CpG island methylation was confirmed by direct sequencing in one tumour (Figure 4B). All *SLIT2* methylated tumours contained unmethylated *SLIT2* alleles that might be attributable to the presence of contaminating normal tissue (tumour samples were not microdissected). *SLIT2* methylation was detected in zero of six normal tissue samples corresponding to the methylated tumours.

To investigate the 4p15.2 allelic status of Wilms' tumours with *SLIT2* methylation, we typed six methylated tumours for LOH at D4S1546. None of three informative tumours demonstrated D4S1546 allele loss.

#### Methylation of *SLIT2* and other cancer genes in primary Wilms' tumours

To investigate the relationships between *SLIT2* promoter methylation and *de novo* methylation of *RASSF1A* and *CASP8*, we compared the frequencies of *CASP8* and *RASSF1A* methylation in tumours with and without *SLIT2* methylation. In tumours with *SLIT2* methylation, *CASP8* and *RASSF1A* were methylated in 43% (six out of 14) and 36% (five out of 14), respectively. In tumours without *SLIT2* methylation, *CASP8* and *RASSF1A* promoter methylation was detected in 39% (nine out of 23) and 70% (16 out of 23), respectively. Thus, although there was no association between *SLIT2* and *CASP8* methylation, there was an inverse relationship between *SLIT2* and *RASSF1A* methylation, although this did not reach statistical significance (*P*=0.09).

#### *SLIT* methylation status and clinicopathological status

The frequency of relapse in Wilms' tumours with *SLIT2* methylation was similar to that without *SLIT2* methylation (21% (three out of 14) and 17% (four out of 23), respectively), and there was no significant association between *SLIT2* methylation and advanced stage tumours (the frequency of stage 3 and 4 tumours in the *SLIT2* methylated and unmethylated groups was 45% (five out of 11) and 63% (12 out of 19), respectively).

### Methylation analysis of *SLIT2* in primary RCC

We detected *SLIT2* promoter methylation in 25% (12 out of 48) primary RCC and in 75% (six out of eight) RCC cell lines (Figure 1C). Promoter CpG island methylation was confirmed by direct sequencing of five clones from RCC cell lines and 2 tumours (Figure 4A). *SLIT2* promoter methylation was also detected in one out of 12 of the matching normal kidney tissue samples for methylated tumours. All RCC with *SLIT2* methylation also contained unmethylated *SLIT2* alleles, which might be attributable to the presence of contaminating normal tissue (tumour samples were not microdissected). Loss of heterozygosity at D4S1546 was not detected in 10 informative RCC with *SLIT2* methylation.

#### *SLIT2* promoter methylation is associated with transcriptional silencing in an RCC cell line

We investigated the possible association between the *SLIT2* promoter region CpG island methylation and *SLIT2* transcript expression in a panel of RCC cell lines (SKRC 18, SKRC 39, SKRC 45, SKRC 47, SKRC 54, KTCL 26,UMRC-2 and 786-0). Cells were treated with the demethylating agent 5-aza-dC for 5 days. Except for SKRC 45 and SKRC 47 (both unmethylated for *SLIT2*), *SLIT2* expression was significantly increased in the kidney tumour cell lines after 5-aza-dC treatment. *GAPDH* expression levels were equal in both 5-aza-dC-treated and untreated cell lines (Figure 3

).

#### Methylation status of *SLIT2* and inactivation of *RASSF1A* and VHL in primary RCC

Previously, we analysed primary RCC for *RASSF1A* methylation and inactivation of the *VHL* tumour suppressor gene. There was no association between *SLIT2* methylation and the presence of *VHL* mutation in clear cell RCC, and the frequency of *RASSF1A* methylation was similar in RCC with and without *SLIT2* methylation (25 and 21%, respectively).

#### *SLIT2* methylation status and clinicopathological status

The frequency of *SLIT2* methylation in clear cell RCC (24%, nine out of 37) was similar to that found in all tumour types. There was no significant association between *SLIT2* methylation status and grade or TNM status.

## DISCUSSION

Previously, we (a) identified *SLIT2* promoter methylation in 53% non-small-cell lung cancer, 36% small-cell lung cancer and 43% of breast cancers, (b) demonstrated that promoter methylation is associated with reversible transcriptional silencing and (c) determined that restoration of *SLIT2* expression suppressed tumour growth in *in vitro* studies (Dallol *et al*, 2002a). Thus having established *SLIT2* as a lung and breast cancer suppressor gene, we have now identified frequent *SLIT2* hypermethylation in paediatric cancers and in RCC and, recently, in 59% of gliomas (Dallol *et al*, 2003). Slit protein binds to the roundabout receptor and acts as a midline repellent to guide axonal development during embryogenesis (Rajagopalan *et al*, 2000; Simpson *et al*, 2000; Wong *et al*, 2001) In humans, three *SLIT* orthologues have been identified, but to date only *SLIT2* has been implicated in cancer. In mice, *Robo1* inactivation produces delayed lung maturation and bronchial hyperplasia (Xian *et al*, 2001) and we have demonstrated *de novo ROBO1* promoter methylation in 19% primary invasive breast carcinomas and 18% primary clear cell RCC, although somatic mutations were not identified (Dallol *et al*, 2002b).

The characterisation of tumour-specific TSG methylation profiles provides insights into the molecular pathology of the tumour and can be used to establish tumour-specific methylation profiles (Esteller *et al*, 2001a). It is anticipated that such profiles will be useful for diagnosis and clinicopathological classification. In colorectal cancer, the identification of a subset of tumours with widespread *de novo* methylation of TSG and mismatch repair genes (‘the methylator phenotype’) (Toyota *et al*, 1999) has been of considerable interest and it is unclear as to whether similar subtypes will be a feature of other cancers.

The molecular pathology of sporadic NB has been investigated extensively by molecular cytogenetic and LOH analysis. Frequent alterations include N-myc amplification (20–25%) and gain of genetic material at 17q23–qter (∼50% of tumours). Neuroblastoma TSGs have been mapped by LOH studies to 1p36 (∼35% in primary tumours show LOH), 11q23 (44%), 14q23–qter (22%) and 3p (15%) (Ejeskar *et al*, 1998; Maris and Matthay, 1999). However, specific gene mutations have not been defined, although we did identify *RASSF1A* methylation in 55% of NBs and that *CASP8* methylation occurs in ∼50% of tumours (Teitz *et al*, 2000; Astuti *et al*, 2001). Caron *et al* (1996) reported 4p allele loss in ∼20% of NB and we have now identified promoter methylation of the 4p15.2 candidate TSG *SLIT2* in 29% of NBs. Thus, epigenetic inactivation of *SLIT2* is a common feature of NB, although less frequent than methylation of *CASP8* and *RASSF1A*. Harada *et al* (2002b) reported that NB patients with CASP8 methylation were older than those without tumour methylation, but to date, *CASP8*, *RASSF1A* or *SLIT2* methylation has not been associated with specific clinicopathological, cytogenetic or molecular features of NB. However, very large studies may be needed to identify significant prognostic correlations in the presence of a large number of potential variables. We did not find clear evidence of a ‘methylator phenotype’, although methylation of *RASSF1A* was more common in tumours with *SLIT2* methylation than in those without *SLIT2* methylation and Harada *et al* (2002b) reported an association between *RASSF1A* and *CASP8* methylation in NB tumours. However, we found no association between *SLIT2* and *CASP8* methylation. The frequency of *SLIT2* methylation in NB was less than that for *RASSF1A* and *CASP8*, but is still a significant finding as other candidate TSGs that demonstrate frequent promoter methylation in some cancers (e.g. *p16INK4A, MGMT, RARâ, DAPK, APC, GSTP1, CDH1* and *CDH13*) are rarely methylated in NB (Harada *et al*, 2002a).

Although *de novo* methylation and silencing of *H19* in Wilms' tumours was first reported some years ago (Steenman *et al*, 1994; Taniguchi *et al*, 1995), epigenetic changes have not been investigated in great detail in Wilms' tumour. Recently, we reported frequent *CASP8* (43%) and *RASSF1A* (56%) promoter methylation in Wilms' tumours and the present study has demonstrated that *SLIT2* methylation represents a further frequent epigenetic change in Wilms' tumours. To date, we have not identified an association between *CASP8*, *RASSF1A* and *SLIT2* methylation in individual tumours, so there is little evidence of a methylator phenotype in a subset of Wilms' tumours. Indeed there was a negative, albeit statistically insignificant, correlation between *RASSF1A* and *SLIT2* methylation. This finding merits further investigation as it could indicate that the simultaneous inactivation of specific TSGs might be disadvantageous in specific cancer types. Although *p16*^*INK4a*^(*CDKN2a*) promoter methylation has been reported in advanced stage Wilms' tumours (Arcellana-Panlilio *et al*, 2000), this trend was not significant in our series, and to date no specific clinicopathological features have been associated with *SLIT2*, *CASP8* and *RASSF1A* methylation. In our Wilms' tumour series, the frequency of *SLIT2* promoter methylation in Wilms' tumours was similar to that for *CASP8* but higher than that for TSGs, which may show frequent promoter methylation in other tumour types, for example, *MGMT* (30%), *NORE1A* (15%), *p14*^*ARF*^ (15%), p16^INK4a^ (10%), *DAPK* (11%), *CRBP1* (9%), *RARB2* (0%), *CDH13* (0%) and *CDH1* (3%) (Morris *et al*, 2003).

Renal cell carcinoma is the most common adult tumour and the majority (∼75%) of RCC are classified as clear cell RCC, with papillary being the most frequent nonclear cell histopathological subtype (∼15% of all cases) (Storkel and van den Berg, 1995). The most frequent genetic change in RCC is somatic inactivation of the *VHL* TSG (usually be mutation and loss, but promoter methylation may also occur), although *VHL* inactivation is specific for clear cell RCC (Foster *et al*, 1994; Gnarra *et al*, 1994; Clifford *et al*, 1998). Combining the results of the current study with previous investigations, frequent epigenetic changes (⩾20%) in RCC include promoter methylation of *RASSF1A*, *TIMP3*, *DAPK*, *SLIT2, MT1G* and *GSTP1* (Esteller *et al*, 2001a; Nojima *et al*, 2001, Dreijerink *et al*, 2001; Morrissey *et al*, 2001, Morris *et al*, 2003). In contrast, promoter methylation not at (or rarely at) SDHB, RARB2, *p16*^*INK4a*^ and *CDH13* is uncommon (<5%). To date, apart from VHL, none of the epigenetic changes in RCC have been associated with specific clinicopathological features.

The failure to detect an association between clinicopathological stage and *SLIT2* methylation status could indicate that SLIT2 methylation is an early event in tumorigenesis. In tumours such as colorectal cancer, where there is a well-validated adenoma–carcinoma sequence, it is possible to define the genetic changes associated with different stages of tumorigenesis. However, in sporadic cases of NB, Wilms' tumour and RCC, there is generally no well-defined pathway from precursor lesion to tumour (although nephroblastomatosis may be present in patients with Beckwith–Widemann syndrome and ‘early lesion RCC’ has been described in von Hippel-Lindau disease). Hence, we are unable to precisely define when SLIT methylation occurs in the pathogenesis of these tumours. However, it is known that TSG inactivation may be an early event in tumorigenesis. Thus, methylation may be the ‘second hit’ in familial cancer syndrome tumours (Prowse *et al*, 1997, Esteller *et al*, 2001b). Furthermore, in sporadic and familial adenomatous polyposis coli, TSG CpG island methylation may be detected in the earliest precursor lesion in colorectal carcinogenesis, aberrant crypt foci (Chan *et al*, 2002). Similarly, Zöchbauer-Müller *et al* (2001) suggested that TSG methylation may be a preneoplastic change in non-small-cell lung cancer. We have analysed previously *RASSF1A* promoter methylation status in normal, ductal-carcinoima-*in situ* (DCIS) and breast cancer trios. *RASSF1A* promoter hypermethylation was detected in 65% of invasive cancers and in 42% of corresponding DCIS but in none of the normal breast samples (Honorio *et al*, 2003). In all, 30% of DCIS without invasive breast cancer also underwent *RASSF1A* promoter hypermethylation, suggesting that inactivation of *RASSF1A* by CpG island methylation is an early event in breast tumorigenesis. Preliminary unpublished data also reveal *SLIT2* methylation in DCIS samples (RE Dickinson and F Latif, unpublished). Thus, there is evidence that *SLIT2* hypermethylation can be implicated in early tumorigenesis.

In breast and lung cancers, TSG promoter methylation *SLIT2* appears to resemble TSGs such as *RASSF1A,* as epigenetic inactivation is more frequent than somatic mutations. *RASSF1A* methylation has been reported in a wide range of human cancers. We have demonstrated that *SLIT2* methylation is common in paediatric and adult cancers, and further analysis of additional tumour types seems indicated. Frequent 4p allele loss has been reported in cancers that demonstrate *SLIT2* methylation such as lung, breast and NB, and also in cancers in which *SLIT2* methylation status has not been investigated including colorectal, bladder and head and neck cancers (Knowles *et al*, 1994; Rosin *et al*, 1995; Gryfe *et al*, 1997; Pershouse *et al*, 1997; Yustein *et al*, 1999; Girard *et al*, 2000). During development, the *SLIT2* protein functions as a secreted chemorepellent so that restoration of *SLIT2* function by reversal of epigenetic inactivation or administration of *SLIT2* agonists might provide novel therapeutic opportunities for human cancers.
